# The Texas State Medical Association

**Published:** 1898-03

**Authors:** 


THE TEXAS STATE MEDICAL ASSOCIATION.—Let it be borne in mind that the annual meeting of the Texas State Medical Association will be held in Houston, beginning on the fourth Tuesday in April, prox. A splendid program has been provided for the occasion, and a very large attendance is expected.

We had hoped to be able to publish the program in this issue, but the secretary, Dr. West, has not been able to get it ready. In our April number we will publish the program in full, together with all necessary information for the guidance of delegates and those who wish to become members.

For several years there has been a slow decadence in the Association, due mainly, we believe, to the injudicious choice of places of meeting. For the last few years the meetings have been held on the extreme borders of the State, at least, at points too remote for most of the members to attend. For instance: Last year the meeting was held at Paris, on the extreme northern border of the State, and the result was, not one out of twenty of the members residing in the southern portion of the State was able to attend. We, living at Austin, were twenty-four hours on the road, both going and coming. That is an awful bore. A few years before that, the meeting was held at Tyler, and those two meetings were the smallest ever known in the history of the Association. Houston is easily accessible from all points, and such concessions will be made in rates as to induce all to attend. Those desiring to join should bring their diploma with them.

